# Supplementary material for: Brain‐targeted stem cell gene therapy corrects mucopolysaccharidosis type II via multiple mechanisms
Source: EMBO Mol Med. 2018 Jun 8;10(7):e8730. doi: 10.15252/emmm.201708730 (PMC6034129; doi:10.15252/emmm.201708730)
Supplement: Supplementary file 1 — Appendix [file EMMM-10-e8730-s001.pdf]

Brain targeted stem cell gene therapy corrects Mucopolysaccharidosis type II via multiple mechanisms

Hélène F. E. Gleitz[1], Ai Yin Liao[1], James R. Cook[1], Samuel F. Rowlston[1], Gabriella M. A. Forte[1], Zelpha D'Souza[1], Claire O'Leary[1], Rebecca J. Holley[1], Brian W. Bigger[1]*

Appendix

Table of contents:

Appendix Figure 1 Page 2

Appendix Figure 2 Page 4

Appendix Figure 3 Page 5

Appendix Figure 4 Page 6

Appendix Figure 5 Page 7

Appendix Table 1 Page 8

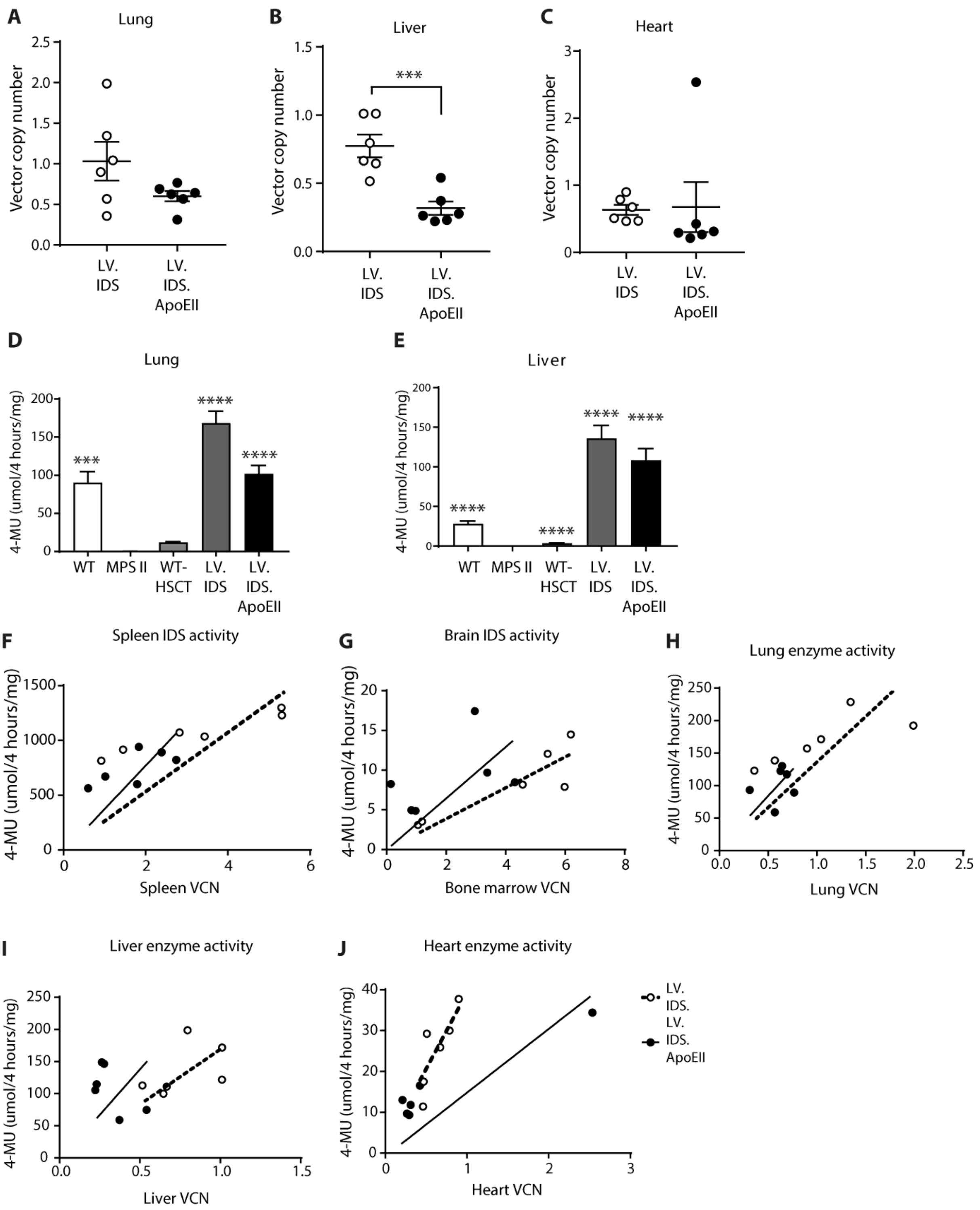

Appendix Figure 1 - LV.IDS and LV.IDS.ApoEII produce supraphysiological levels of IDS enzyme in lung and are detected in additional peripheral organs.

A-C Vector copy number was measured in lung (A), liver (B) and heart (C).

D-E: IDS enzyme activity were measured in organs taken at 8 months of age, including lung (D) and liver (E) from control and treated mice, *n*=6/group. Data was log transformed prior to statistical analysis.

F-J VCN to enzyme activity correlation in individual mice in (F) spleen, (G) BM/brain, (H) lung, (I) liver and (J) heart. Lines showing the linear regression are shown.

Data are shown as mean ± SEM with one way-ANOVA or T-test performed as appropriate, ***=p<0.001, ****=p<0.0001 vs. MPS II, other comparisons are indicated by brackets.

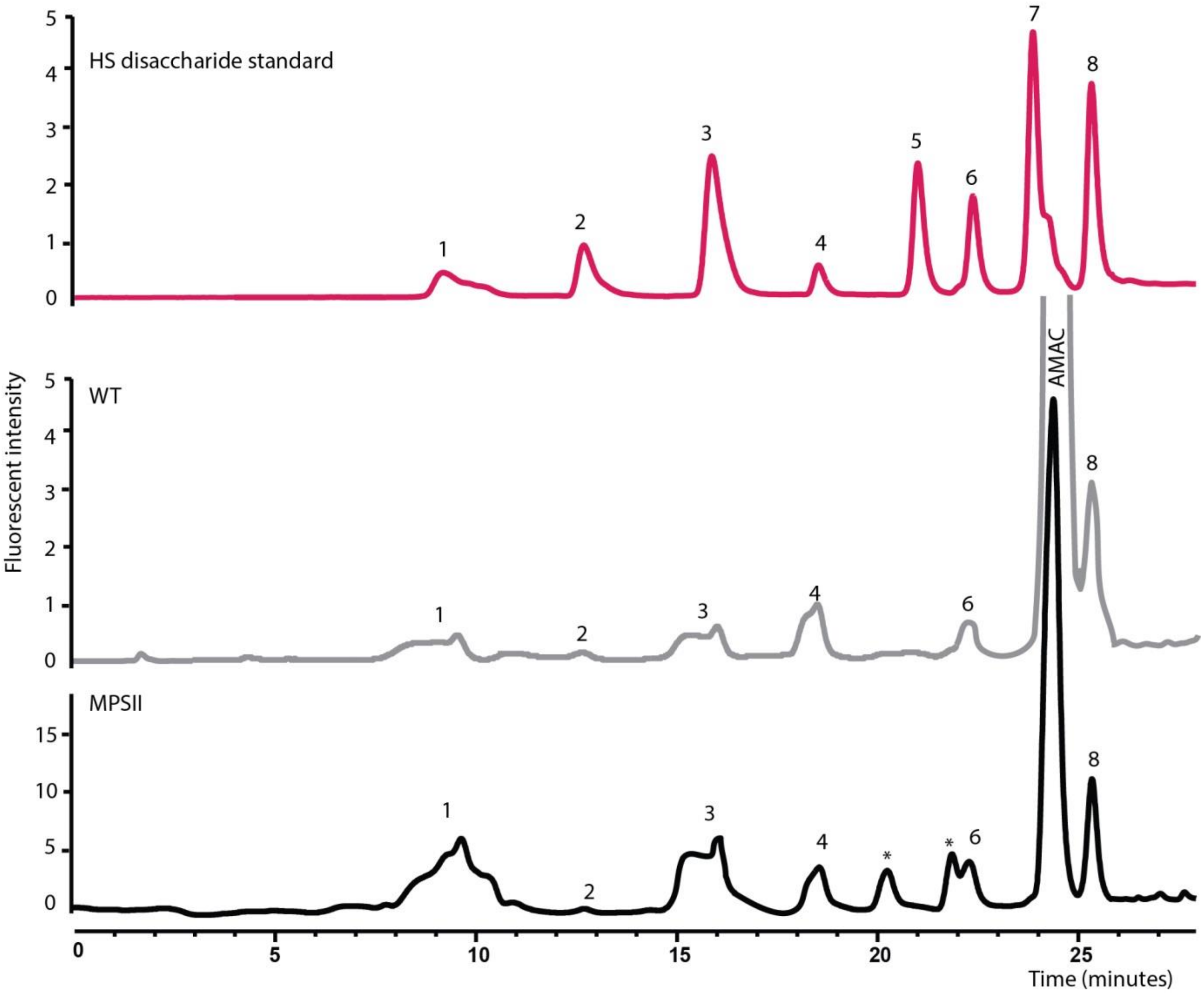

Appendix Figure 2 – HPLC heparan sulfate profiles.

HPLC profiles for 8 disaccharide AMAC-labelled standards (red) or GAGs wild-type (grey) or MPSII (black) following heparinase I/II/III digestion and AMAC labelling. Disaccharides are numbered according to comparison to known standards: 1, HexA(2S)-GlcNS(6S); 2, HexA-GlcNS(6S); 3, HexA(2S)-GlcNS; 4, HexA-GlcNS; 5, HexA-GlcNAc(6S); 6, HexA-GlcNAc; *, uncharacterized (MPSII specific) and peak at ~24 minutes is free AMAC remaining following labelling.

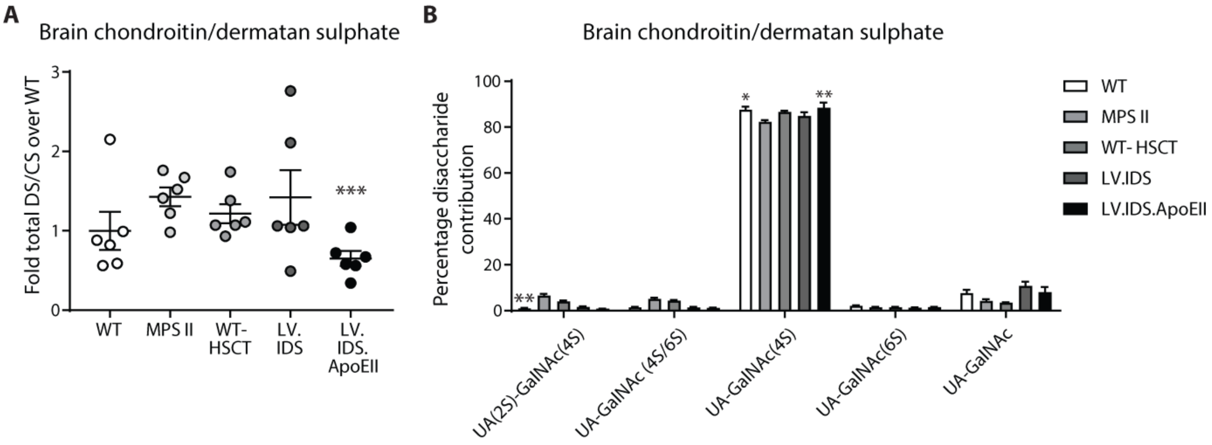

Appendix Figure 3 - LV.IDS.ApoEII normalizes chondroitin/dermatan sulfate levels in the brain of 8-months-old MPS II mice.

A Total relative amounts of CS/DS from control and treated mice in the brain.

B Compositional disaccharide analysis of CS/DS from control and treated mice in the brain.

Data are shown as mean ± SEM, *n*=6/group, * = p<0.05, ** = p<0.01, *** = p<0.001 vs. MPS II, other comparisons are indicated by brackets.

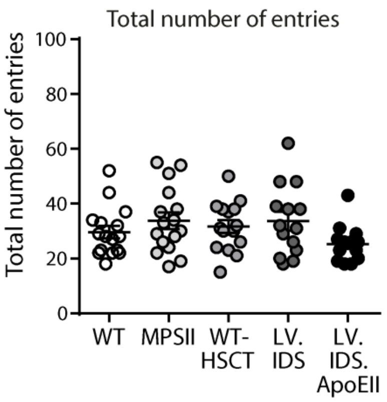

Appendix Figure 4 - Locomotor activity in the Y-maze.

A Total number of arm entries recorded as a proxy measure of locomotor activity during the 10 minute-test in the Y-maze, n=12-16.

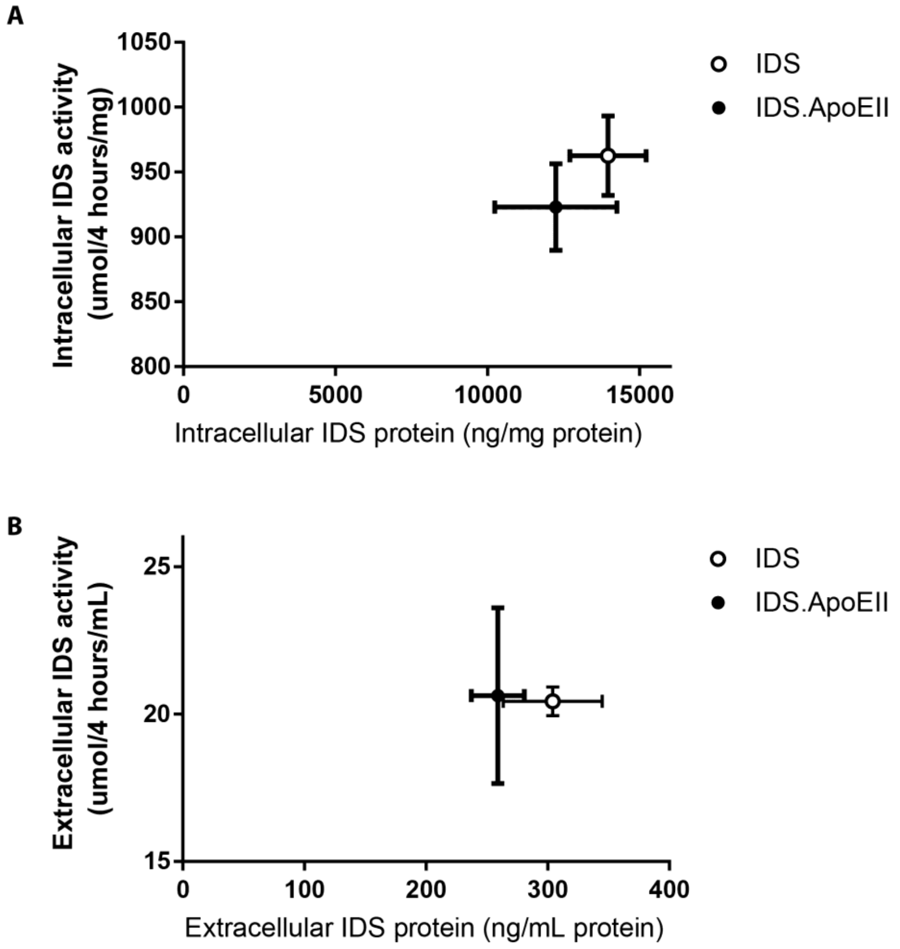

Appendix Figure 5 – IDS and IDS.ApoEII show equivalent levels of enzyme activity per unit of protein *in vitro*.

A Intracellular IDS activity versus IDS protein in the human microglial cell line CHME3 cells after transfection with 2µg plasmid DNA of either LV.IDS or LV.IDS.ApoEII and measured 48 hours post-transfection, *n*=4.

B Extracellular IDS activity versus IDS protein released into the media after transfection of the human microglial cell line CHME3 cells with 2µg plasmid DNA of either LV.IDS or LV.IDS.ApoEII and measured 48 hours post-transfection, *n*=4.

Appendix Table 1 - AMAC correction factors for the detection of HS and CS/DS disaccharides

Disaccharide	AMAC correction factor
ΔUA(2S)-GalNAc(4S,6S)	1.38
ΔUA(2S)-GalNAc(4S)	1.35
ΔUA-GalNAc(4S,6S)	1.50
ΔUA-GalNAc(4S)	1.43
ΔUA-GalNAc(6S)	1.35
ΔUA-GalNAc	1.40
ΔUA(2S)-GlcNS(6S)	1.25
ΔUA-GlcNS(6S)	1.13
ΔUA(2S)-GlcNS	1.00
ΔUA-GlcNS	1.04
ΔUA-GlcNAc(6S)	1.13
ΔUA-GlcNAc	1.08
